# A novel miRNA biomarker panel associated with mortality in pediatric patients with ARDS

**DOI:** 10.1186/s12931-021-01761-5

**Published:** 2021-06-04

**Authors:** Lisa K. Lee, Mansoureh Eghbali, Anil Sapru

**Affiliations:** 1 Department of Anesthesiology and Perioperative Medicine, David Geffen School of Medicine at UCLA, 757 Westwood Plaza, Suite 3325, Los Angeles, CA 90095 USA; 2 Division of Pediatric Critical Care, Department of Pediatrics, David Geffen School of Medicine at UCLA, 10833 Le Conte Ave, MDCC 488, Los Angeles, CA 90095 USA

**Keywords:** miRNA, ARDS, Pediatrics, Biomarkers

## Abstract

We identified a novel microRNA biomarker panel consisting of 6 microRNAs predicting mortality in pediatric acute respiratory distress syndrome patients. Each of the identified mRNA have potential mechanistic importance in acute respiratory distress syndrome and may lead to the development of pharmacologic targets.

## To the Editor:

Acute respiratory distress syndrome (ARDS) is a syndrome characterized by increased permeability in the alveolar-capillary junction resulting in pulmonary edema and hypoxemia in the absence of left heart failure [1]. In the pediatric population worldwide, mortality from ARDS is estimated to be 18% [2] and most survivors have a significant long term decline in performance scores and quality of life [3]. However, there are no specific therapies other than use of lung-protective ventilation and supportive care [4]. Therefore, there is an urgent need to develop targeted therapies. There has been increasing interest in the role that microRNAs (miRNAs) may play in the pathogenesis of ARDS recently [5]. MiRNAs are short non-coding RNA that inhibit post-transcriptional expression of their target genes [5]. The stability of miRNA in plasma is well-documented [5]. In addition to their potential as therapeutic targets, circulating miRNAs have recently emerged as biomarkers for prognostication in several inflammatory diseases, including ARDS [5].

We identified a panel of circulating miRNA in pediatric ARDS patients that are differentially-expressed between survivors and non-survivors. We performed miRNA profiling on 63 pediatric patients (43 survivors and 20 non survivors) who met American-European Consensus Conference Criteria for ARDS [6] and required positive pressure ventilation. These patients were part of a cohort of 235 pediatric patients enrolled in a multi-center prospective study of ARDS biomarkers, aged 30 days to 18 years, from 5 pediatric intensive care units (ICUs) at major children’s hospitals across the country. Subjects were excluded if they had limited goals of care. Enrolled subjects were included in this study if they had sufficient leftover plasma for miRNA extraction. IRB approval was obtained for the use of stored plasma samples prior to initiation of this project.

The plasma samples collected within 24 h of ICU admission were matched two survivors to every non-survivor. The samples were also matched for mechanism of lung injury (direct vs. in-direct), sex and age within 1 month for ages below one year, within 6 months for ages 1–2 years, within 12 months for patients aged 5–10 years and within 24 months for ages over 11 years. The primary outcome of non-survival was defined as in-hospital mortality prior to discharge. Total RNA enriched in small RNA was extracted from the plasma using the Qiagen miRNeasy Plasma/Serum Kit. miRNA profiling of the RNA samples performed using the Nanostring nCounter Human miRv3 Assay. Means with standard deviations and medians with quartiles were calculated for baseline patient characteristics. Differences between survivors and non-survivor groups were tested using t-test and chi-square test for continuous data and discrete data, respectively. Multivariable logistic regression was used to evaluate the association between age, gender, cause of ARDS, Pediatric Risk of Mortality (PRISM-3) score [7] on day 1 of ICU admission, and expression levels of miRNA with mortality. Area Under the Receiver Operating Characteristic Curve (AUROC) analysis was used to evaluate the predictive ability of 4 logistic regression models for mortality: PRISM-3 score alone, the miRNA panel alone, PRISM-3 + miRNA panel, and PRISM-3 + miRNA panel + age + gender + ARDS cause. Normalization of the raw miRNA expression counts was performed using the nSolver 4.0 software. DESeq2 package in ‘R’ was used for differential expression analysis and the pROC package was used for AUROC analysis. Benjamini–Hochberg procedure with false discovery rate set at 10% was used to correct for multiple comparisons. ClustVis Tools was used for data visualization. No statistically significant differences in baseline patient characteristics were detected between the survivor and non-survivor groups (Table 1).

**Table 1:** Patient characteristics

	All patients (n = 235)	Survivors (n = 43)	Non-survivors (n = 20)	p-value
Age, years (IQR)	4.1 (1.0–11.5)	8.9 (1.6–13.6)	10.4 (2.2–12.7)	0.92
Gender, male (%)	125 (53.2)	27 (62.8)	15 (75.0)	0.34
ARDS cause				0.40
Pulmonary cause (%)	139 (59.1)	27 (62.8)	14 (70.0)	
Non-pulmonary cause (%)	94 (40.0)	16 (37.2)	5 (25.0)	
Race (%)				0.66
African-American	17 (7.2)	4 (9.3)	1 (5.0)	
Asian/Pacific Islander	15 (6.4)	5 (11.6)	2 (10.0)	
Caucasian	160 (68.1)	19 (44.2)	6 (30.0)	
Latino	84 (35.7)	11 (25.6)	8 (40.0)	
Other	43 (18.3)	4 (9.3)	3 (15.0)	
Type of respiratory support (%)				0.45
Mechanical ventilation		36 (83.7)	16 (80.0)	
High frequency oscillation		1 (4.7)	2 (10.0)	
CPAP/BiPAP		5 (11.6)	1 (5.0)	
High flow nasal cannula		1 (4.7)	1 (5.0)	
PRISM score (IQR)	12 (7–20)	12 (5.5–17)	15 (9–19.25)	0.11
Vasopressor use (%)		12 (27.9)	9 (45.0)	0.18

**Fig. 1:**
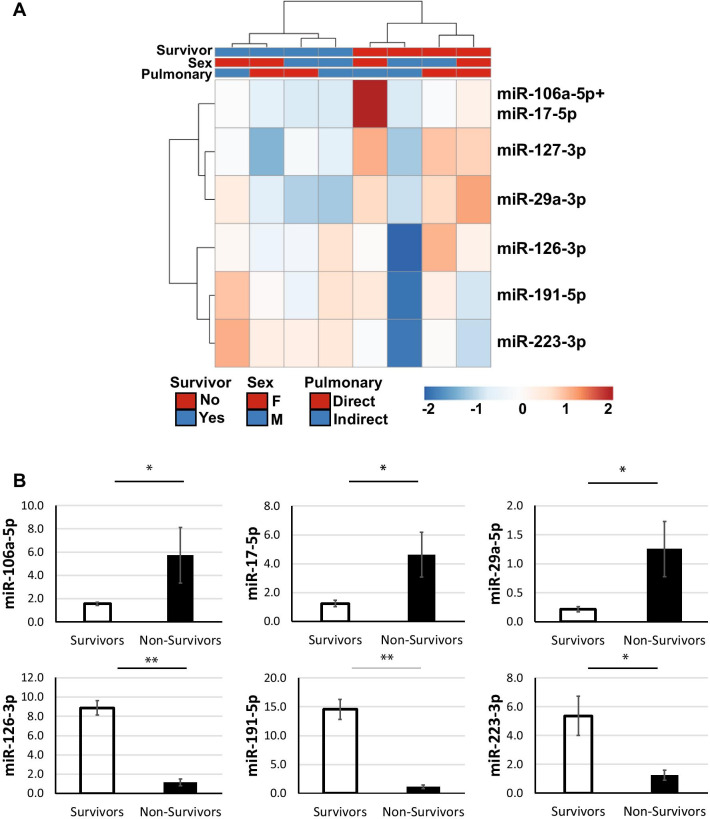
**A** Heatmap of miRNAs found to be differentially expressed in pediatric survivors and non-survivors of ARDS. The original values were log-transformed. Columns with similar annotations (i.e. male, non-survivors with a pulmonary cause of ARDS or female, non-survivors with non-pulmonary cause of ARDS) were collapsed by taking median inside each group. Both rows and columns are clustered using correlation distance and average linkage. **B** Quantitative PCR validation of the individual miRNAs by Taqman assays. Eight samples were used for validation—four samples from survivors and four samples from non-survivors. Increased expression of miR-126-3p, miR-191-5p and miR-223-3p and decreased expression of miR-106a-5p, miR-17-5p and miR-29a-5p in the survivors compared the the non-survivors group were similar to the results obtained by Nanostring. *p < 0.05, **p < 0.001

**Fig. 2:**
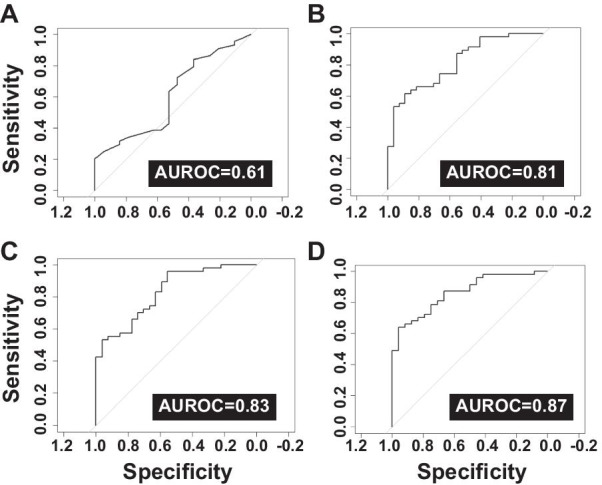
The ability of the biomarker panel to predict mortality was evaluated using AUROC analysis of the multivariable logistic regression models composed of PRISM-3 score only (**A**), the miRNA panel only (**B**), PRISM-3 Score + miRNA panel (**C**), and PRISM-3 Score + miRNA panel + Age + Gender + Cause of ARDS (**D**)

Our plasma miRNA profiling revealed 6 miRNA that were differentially-expressed between pediatric survivors and non-survivors of ARDS (Fig. 1A). Expression of miR-106a-5p + miR-17-5p, miR-127-3p and miR29a-3p were significantly higher, while the expression of miR-126-3p, miR-191-5p, and miR-223-3p were significantly lower among non-survivors compared to survivors. miR-223-3p provided the best differentiation between the survivor and non-survivor group. We validated increased expression of miR-106a-5p, miR-17-50, and miR-29a-3p and decreased expression of miR-126-3p, miR-191-5p, and miR-223-3p in non-survivors vs. survivors in our cohort by PCR (Fig. 1B). However, we were unable to validate expression of miR-127-3p by PCR because this miR was expressed at very low level, below the limit of PCR detection. Next, we evaluated our biomarker panel’s predictive ability for mortality compared to PRISM-3. In our cohort, the PRISM-3 score had an AUROC of 0.61 (95% CI 0.45–0.76, Fig. 2A), whereas the six miRNA that were differentially expressed between survivors and non-survivors had an AUROC of 0.81 (95% 0.72–0.91, Fig. 2B). When the biomarker panel and PRISM score were combined together, its predictive ability for mortality improved with an AUROC of 0.83 (95% CI 0.74–0.92), and addition of age, sex and cause of pulmonary injury (direct vs. indirect) further improved AUROC to 0.87 (95% CI 0.78–0.95, Fig. 2 C, D) We have, therefore, identified a panel of miRNA important in the pathogenesis of ARDS which may also be useful in the prediction of mortality.

Of the six differentially-expressed miRNAs in our study, miR-17-5p, miR-126-3p, miR-127-3p and miR-223-3p were found to be important in other adult studies or experimental animal models of ARDS. miR-17 expression is increased in leukocytes of adult patients with ARDS. Its release is known to promote macrophage migration and recruitment in mouse models of ARDS [8]. miR-126-3p has been suggested to be important in maintaining lung endothelial and epithelial barrier integrity and its expression is downregulated in animal models of ventilator induced lung injury [9]. miR-127-3p is known to play a role in lung development [10] and has been previously reported to be decreased in the lung, bronchoalveolar lavage fluid, and serum in rat and mouse ventilator-induced and intra-tracheal LPS lung injury models of ARDS [10]. Interestingly, we found that miR-223-3p expression was decreased among non-survivors in our cohort. Increased expression of miR-223-3p has previously been reported in the leukocytes and plasma of ARDS patients compared to healthy controls [11] and critically-ill non-ARDS patients [12], suggesting that baseline levels of miR-223 in the absence of ARDS remain low, but expression is increased in ARDS. However, miR-223-3p has been suggested to be protective in murine models of ARDS [13] and associated with decreased expression of IL-6 and IL-1β in macrophages, possibly via targeting STAT3 [14]. This is consistent with the results we have observed in our study where the survivors had higher expression levels of miR-223 compared to the non-survivors.

The other two miRNA that were differentially-expressed in our cohort, miR-29a-3p and miR-191-5p have not been previously established to be of importance in ARDS. However, miR-29a is known to play an important role in pulmonary fibrosis [15], and thus may also be of significance in the fibrotic phase of ARDS. Remarkably, decreased expression of miR-191-5p has been demonstrated in patients with severe sepsis compared to those with severe SIRS [16], corresponding to the decreased expression of miR-191-5p in the non-survivors of ARDS in our cohort.

In conclusion, we identified a novel miRNA biomarker panel consisting of 6 miRNAs predicting mortality in pediatric ARDS patients. Each of the identified mRNA have potential mechanistic importance in ARDS. Additional research into these miRNAs may lead to the development of pharmacologic targets.

## Data Availability

The datasets used and/or analyzed during the current study are available from the corresponding author on reasonable request.

## Abbreviations

ARDS: Acute respiratory distress syndrome
miRNA: MicroRNA
ICU: Intensive care unit
PRISM: Pediatric risk of mortality
AUROC: Area under the receiver operating characteristic curve
